# Diagnostic Difficulties and Treatment Challenges of a Young Patient With Severe Acute Psychosis and Complete Recovery

**DOI:** 10.7759/cureus.23744

**Published:** 2022-04-01

**Authors:** Jagoda Siembida, Saaduddin Mohammed, Mariam Chishty, Luba Leontieva

**Affiliations:** 1 Psychiatry, State University of New York Upstate Medical University, Syracuse, USA

**Keywords:** covid-19 induced psychosis, clinical psychiatry, antipsychotic medication, schizophrenia and other psychotic disorders, cannabis induced psychosis, first episode psychosis

## Abstract

First break psychosis in young adults is sometimes presented as a dichotomous model of organic or substance-induced etiology or a primary psychiatric disorder on the schizophrenia spectrum and related disorders. In this case of a young adult with a typical age of onset for psychotic symptoms also presenting with cannabis use, excessive vaping, history of COVID-19 illness, pineal cyst, and extreme elevation of blood pressure, the diagnostic certainty decreases. Increased risk of progression to schizophrenia in individuals with cannabis use disorder and genetic loading has been extensively reported in the literature. Clinicians may face significant diagnostic and treatment challenges when managing a patient with severe psychotic symptoms. For the clinicians acutely managing such patients facing these exact questions of unknown certainty in progression to full-blown schizophrenia, we highlight a case of severe acute psychosis and complete recovery on a first-generation antipsychotic and mood stabilizer.

## Introduction

We present the case of a 21-year-old highly-functioning female college student of high socioeconomic background admitted with first break psychosis. Her presentation proved challenging given her unexplained hypertension, the recent history of weight loss, seemingly incidental finding of pineal gland abnormality on brain imaging, and atypical response to a second-generation antipsychotic with hypersexuality. Her clinical picture was further complicated by a history of excessive cannabis use and vaping in the months leading up to her psychosis and COVID-19 infection about a year prior to that. With strong genetic loading for schizophrenia based on family history, the working diagnosis included schizophreniform disorder with good prognostic factors. Her slow response to treatment with ultimate resolution of psychotic symptoms at the end of hospitalization challenges the initial working diagnosis of the schizophreniform disorder and portrays a likelihood of substance-induced psychosis or psychosis due to a general medical condition as the main differential. She improved on a first-generation antipsychotic, haloperidol, and the mood stabilizer valproic acid and returned to pre-morbid functioning, resuming college education several weeks after discharge from the hospital. We highlight the patient's one-month hospitalization, challenges in diagnosis and treatment, and review literature to allow clinicians to recognize diagnostic difficulties in first break psychosis while exploring organic etiology. 

## Case presentation

A 21-year-old Caucasian highly-functioning female college student with no formal past psychiatric history presented to an academic hospital's emergency room for evaluation of a four-day history of paranoia and disorganized behavior. In the days leading up to the emergency room presentation, the patient frantically called her family members, notifying them of feeling starved, having no money, and suffering from insomnia. That same night, she shared news of winning millions of dollars and was preoccupied with a famous financial figure. A concerned roommate called emergency services after the patient was observed talking to herself and yelling for help randomly in their apartment. The patient was reportedly evaluated at an outside hospital's emergency room and discharged shortly after the initial presentation. The patient's family members rushed to locate the patient. Once reunited, they presented to another outside hospital after the patient ran outside screaming for help in an erratic manner.

In the emergency room, the patient presented as labile, delusional, and exhibited ideas of reference. The psychiatry consultant determined the patient exhibited poor reality testing and was internally preoccupied. She attempted to elope from the emergency room and required chemical sedation with haloperidol 5 mg intramuscularly and lorazepam 2 mg intramuscularly. The patient was subsequently admitted to an outside hospital's inpatient psychiatric unit for psychosis. The patient had a brief, one-day hospitalization where she was described as bizarre, laying down on the hospital floor, and walking into peers' rooms, reporting she was feeling anxious. She required hydroxyzine 25 mg oral and olanzapine 5 mg oral for her bizarre behavior. She was later observed on that same day to be unresponsive verbally, "cool and clammy," with an elevated blood pressure of 176/107 mmHg. A rapid response code was called, and the patient was transferred to medicine's service for concern for acute encephalopathy and hypertensive emergency, where she was admitted for a total of two days. Amlodipine and lisinopril were initiated for uncontrolled hypertension. Amlodipine was increased from 2.5 mg to 5 mg, and she was continued on lisinopril 10 mg. The patient required a third antihypertensive, hydralazine, twice during the admission, once at 10 mg and the second dose at 25 mg. The bizarre behavior and paranoia persisted, necessitating a one-time dose of ziprasidone 5 mg intramuscularly for agitation, and then risperidone 0.5 mg as needed for anxiety/agitation in addition to hydroxyzine 50 mg. Melatonin 5 mg and trazodone 25 mg were administered for sleep.

A workup for hypertension was pursued, including an evaluation for secondary causes. A CT of the head was negative for acute intracranial abnormality. Chest X-ray was negative for coarctation of the aorta or any acute findings. Initial EKG revealed left ventricular hypertrophy with no arrhythmia. An echocardiogram revealed no abnormalities. The patient's tall and thin appearance concerned the team as her BMI was <19. A registered weight from an unrelated encounter two months prior was notable for a 4.5 kg weight loss.

Upon stabilization of the patient's blood pressure, the patient was re-admitted to inpatient psychiatry. She continued to demonstrate severe psychotic symptoms and affective instability. Depakote was initiated and titrated to 500 mg BID with good effect. Valproic acid plasma trough levels drawn six days apart were 65 and 63. For several days during the beginning of admission, the patient required daily agitation management consisting of haloperidol 5 mg, lorazepam 2 mg, and diphenhydramine 50 mg PO. The patient displayed slowing, and therefore neuroleptic-induced negative symptoms were considered, as was psychosis in general. There was a concern for adverse effects from dopamine antagonism, and therefore haloperidol PRN (as needed) was discontinued. Severe psychotic symptoms persisted, and with little evidence to suggest an improvement, an atypical antipsychotic agent was initiated while haloperidol was discontinued.

While on olanzapine, the patient developed severe and distressing erotomaniac delusions, accusing her father of raping her, and disorganized behavior with attempts at partial nudity that was out of context to the situation. This behavior was ego-dystonic to the patient. As this behavior was absent during the haloperidol trial, we cross-tapered olanzapine to restart haloperidol, given higher dopamine-2 (D2) blockade. The patient started improving on haloperidol 5 mg BID with divalproex sodium 500 mg BID, as evidenced by the resolving hypersexuality and psychotic symptoms. The severe symptoms prompted further investigation and concern for the patient's prognosis as the severity and acuity appeared to coincide with a classic first break psychosis. Psychological testing (the Thematic Apperception Test [TAT] and the Minnesota Multiphasic Personality Inventory-2 [MMPI-2]) was pursued and suggestive of schizophreniform disorder.

**Figure 1:**
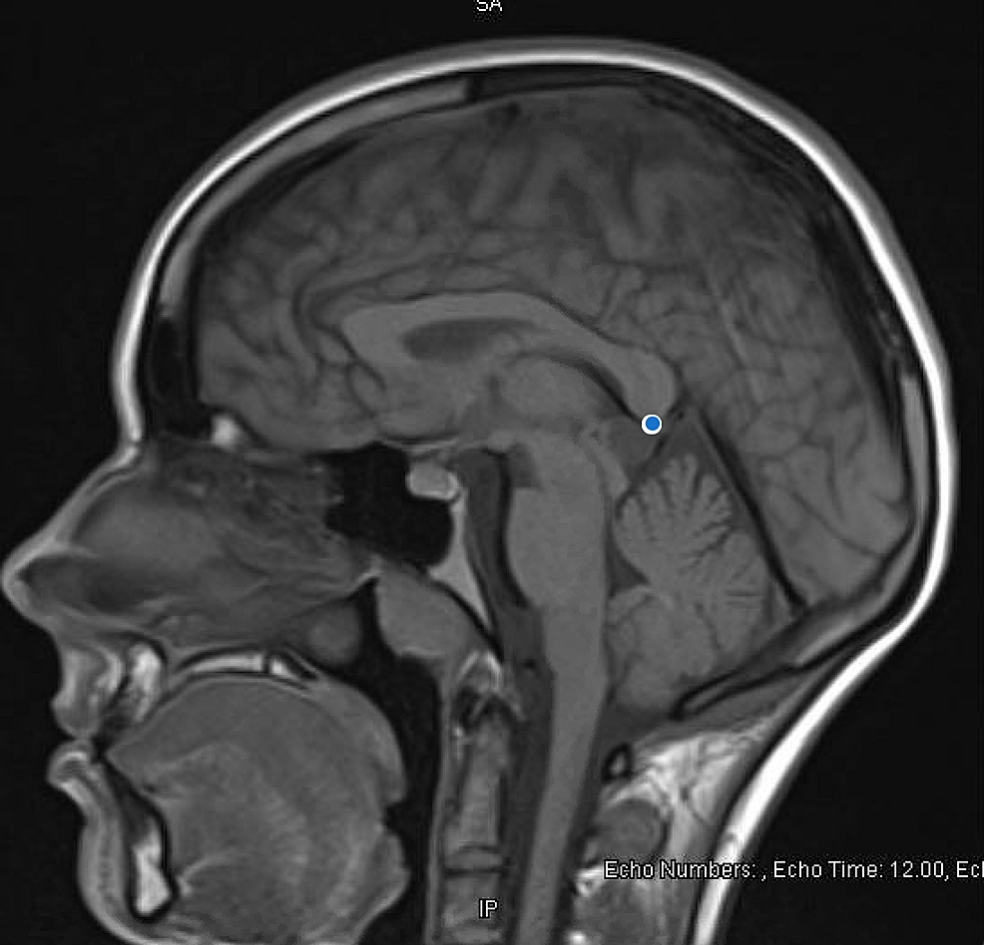
MRI of the brain without contrast sagittal view showing pineal gland cyst Radiologic report:* lesion within the region of the pineal gland, which is T1 hypointense and T2 hyperintense, measuring 1.3 x 1.3 cm, likely consistent with a pineal cyst.* A blue dot marks the area of interest.

Extensive organic workup was pursued for first break psychosis with initial higher suspicion for organic etiology given unexplained hypertension, prompting concern for anti-N-methyl D-aspartate (anti-NMDA) receptor encephalitis. HIV Ag/Ab screen, NMDA IgG, vitamin D, folate, vitamin B6, B12, B1, heavy metal profile, phosphorus, and magnesium levels were all normal. Table 1 summarizes labs pertinent to our investigation. Brain imaging mentioned previously (CT of the head) was negative for intracranial pathology. An MRI of the brain (see Figure 1) was obtained, which was notable for pineal gland cyst and occipital venous anomaly, determined to be incidental after consultation with neurosurgery. 

**Table 1:** Summary of labs pertinent to the workup of organic psychosis Abnormal values are in bold. NMDA IgG <1:10 indicates that antibodies to NMDA were not detected. NMDA - N-methyl D-aspartate; TSH - thyroid-stimulating hormone

Lab	Value
Aldosterone/renin ratio	
Aldosterone, blood	30.5 ng/dL
Renin activity	16.178 ng/mL/hr
Aldosterone/renin ratio	1.9
Drugs of abuse, urine	
Amphetamine	Negative
Benzodiazepine	Negative
Cannabinoids	Positive
Cocaine	Negative
Methadone	Negative
Opiates	Negative
Oxycodone	Negative
Fentanyl	Negative
Folate	14.30 ng/mL
Heavy metal profile	
Lead	<1 ug/dL
Arsenic	11 ug/L
Mercury	2.2 ug/L
HIV Ag/Ab screen	Non-reactive
Magnesium	2.2 mg/dL
NMDA IgG	<1:10
Phosphorus	4.2 mg/dL
TSH	0.845 u[IU]/mL
Vitamin B1	120.2 nmol/L
Vitamin B6	20.0 ug/L
Vitamin B12	886 pg/ml
Vitamin D	41 ng/mL

The patient remained on the antihypertensives and continued to suffer from persistent hypertension throughout admission. Lisinopril was increased to 40mg. Workup of less common secondary causes of hypertension was pursued due to the patient's young age, low BMI, and psychotic symptoms. An interesting aspect of her medical history is COVID-19 infection less than a year from symptom onset and its unclear relation to hypertension. Initial hypertension on admission was deemed secondary to psychotic agitation with underlying primary hypertension. Mid-admission renin, aldosterone, and renin/aldosterone ratio was notable for both renin and aldosterone elevation. However, this was inconclusive due to the patient being on antihypertensives at the time of the tests. Elevated renin and aldosterone may be due to antihypertensives. A CT of the abdomen and pelvis was negative for adrenal abnormalities. The patient's blood pressure eventually stabilized, and the patient tolerated discontinuation of lisinopril and reduction in amlodipine dose to 5 mg daily.

Regarding the patient's history of weight loss, her electrolytes were monitored for refeeding syndrome. The patient was severely malnourished and underweight with a recent 15 lbs unintentional weight loss history. The patient's BMI was 15.5 on admission. Her PO intake remained poor throughout hospitalization, and as she approached discharge, PO intake significantly improved, resulting in a 4 kg weight gain.

By the time of discharge, the patient approached her premorbid baseline. The patient's mental status exam was negative for any disorganized behavior, hallucinations, internal preoccupations, or thought disorder. She was referred to a state-funded first break psychosis program and had a total of four follow-up visits over the course of four weeks with our team after hospitalization. One week after discharge, haloperidol was reduced to 2.5 mg twice daily with benztropine 0.5 mg twice daily. On the second follow-up post-discharge, haloperidol was decreased to 2.5 mg nightly, and benztropine was reduced to 0.5 mg nightly. Lisinopril was decreased to 2.5 mg nightly. By her third follow-up visit, she resumed remote college classes and denied any difficulties such as cognitive or psychomotor symptoms. Her energy levels and previously reported excessive salivation resolved with decreased haloperidol dosing. During her final follow-up visit with the team post-discharge, the patient demonstrated the absence of positive symptoms for one month. The psychosis was completely resolved with the last auditory hallucinations experienced one week prior to discharge. The patient made a remarkable recovery and had plans to return to full-time school. Her follow-up care moving forward included the early psychosis program. Diagnostic impression during final follow-up was notable for unspecified psychosis or cannabis-induced psychosis that was resolved.

## Discussion

This case presents several challenges: diagnosis and treatment. From the diagnostic perspective, the differential diagnoses between schizophrenia spectrum disorder, substance-induced psychosis, psychosis due to a general medical condition (GMC; COVID-19 illness) are under consideration. Below we discuss the differential diagnoses and their challenges.

Cannabis-induced psychosis

Alarming trends in cannabis use amongst young adults continue to make headlines while literature review searches yield extensive amounts of data on risks of psychosis with cannabis use. The Centers for Disease Control and Prevention reports cannabis to be the most commonly used illicit substance in the United States [1]. The 2019 National Survey on Drug Use and Health revealed that 35.4% of young adults aged 18 to 25 were past-year marijuana users [2]. According to The National Institute on Drug Abuse, there was a 9% increase in cannabis use over a five-year period in college students from 2014 to 2019 [3]. Recreational cannabis use trends appear to show increasing use of higher potency cannabis (marked by higher delta-9-tetrahydrocannabinol [THC] / cannabidiol [CBD] ratio), one of which is referred to as "sinsemilla" as it contains higher amounts of THC psychoactive substances and minimal CBD [4,5]. "Sativa" is another known psychoactive substance with increasing mention of its relation to psychosis in literature. One particular study determined that 78% of cannabis users with first break psychosis used high potency cannabis compared to 37% of controls [6]. Endocannabinoids as a system interact with dopamine, gamma-aminobutyric acid (GABA), and glutamate, the same neurotransmitters employed in the pathophysiology of psychosis [7].

Severe cannabis use increases the risk of psychotic disorders by nearly four-fold, according to a 2016 meta-analysis where the reported OR was 3.90 [8]. One-quarter of patients presenting with first break psychosis were suggestive of a substance and/or medication-induced etiology [9]. Cannabis-induced psychotic disorder and its progression to schizophrenia has been recognized by many studies. According to the Diagnostic and Statistical Manual-5 (DSM-5) criteria, a primary psychotic disorder rather than a substance-induced psychotic disorder is diagnosed if symptoms persist after at least one month of sobriety from a given substance [9]. In our case and perhaps in many other cases of first break psychosis, multiple factors contribute to diagnostic uncertainty. Some patients also present with strong genetic loading, which increases the risk of primary psychotic disorders, as in our patient. There is already ample evidence of the heritability of schizophrenia. An interesting concept is the shared heritability of psychosis and cannabis use, with variations of the ATK1 gene identified as a risk factor for psychosis in cannabis users [10,11].

During our patient's almost one-month hospitalization, a schizophreniform diagnosis with good prognostic factors was most appropriate at the time of discharge. The differential included a substance-induced psychotic disorder, given her remarkable return to pre-morbid functioning. However, the risk of schizophrenia persists, given her family history. Are we able to predict her progression to schizophrenia? The DSM-5 reports about two-thirds of individuals with schizophreniform disorder will meet the criteria for schizophrenia [9]. A Swedish study of 7,606 individuals with substance-induced psychosis concluded that cannabis-induced psychosis had the highest conversion rate to schizophrenia in addition to genetic predisposition [12]. Another relevant factor pertaining to the cannabis and psychosis link is the theory of reverse causation [13]. In our case, the patient's cannabis use intensified in the weeks leading up to hospitalization. Was the patient attempting to control disturbing symptoms of psychosis which the patient self-identified as anxiety?

Without long-term follow-up of our patient, we are unable to determine if the patient's presentation was solely due to cannabis and primarily drug-induced psychosis. As the patient's length of stay increased due to continued symptoms in the absence of concurrent substance use in the hospital setting, the timeline of symptoms allowed us to diagnose the patient with schizophreniform disorder. Significant psychotic symptoms, delusions, and hallucinations were present for over one month yet less than six months, consistent with DSM-5 criteria [9]. However, the patient's symptoms were ego-dystonic, with a critical appraisal of her condition once she recovered - a sign of good insight and judgment to support good prognostic features.

The evidence to support a picture more consistent with drug-induced psychosis is the four post-hospital follow-ups with our psychiatry team where the patient remained asymptomatic. The patient was also described as stable, non-psychotic on follow-up with neurosurgery about 2.5 months post-hospital discharge per records. Perhaps the diagnostic uncertainties and limitations are related to the timeline of the patient's improvement in symptoms while adhering strictly to DSM-5 criteria. We emphasize psychoeducation and substance use counseling and continue to support these recommendations in the clinical setting to prevent progression to full-blown schizophrenia.

E-cigarette use and psychiatric comorbidity

The CDC reported that in 2019 approximately 20.8% of U.S. adults (50.6 million) used a tobacco product [14]. Cigarettes were the most commonly used tobacco product among adults, and e-cigarettes were the most commonly used non-cigarette tobacco product (4.5%). The highest prevalence of e-cigarette use was among smokers aged 18-24 years (9.3%), with over half (56.0%) of these young adults reporting that they had never smoked cigarettes.

The convenience of newer pod-like devices, the use of nicotine salts to provide higher doses of nicotine with less throat irritation, and the marketing of e-cigarettes as smoking cessation aids all contribute to lower perceived harm and may account for recent increases in use among adults [15]. The most common reasons given for e-cigarette use are for cessation of tobacco cigarettes or health-related concerns [16]. The liquid of the e-cigarette that is heated to produce an aerosol (that patients inhale into their lungs) can contain nicotine, THC, and/or cannabinoid oils [17].

While still a relatively new phenomenon, widespread uptake of e-cigarettes has led to the development of its own range of problems. The link between cannabis use and psychosis is better studied and more established than the link between e-cigarettes and psychosis. Our patient of study, prior to hospital admission with acute psychosis, recently commenced both cannabis and short-term e-cigarette use. We must consider existing literature to learn about the different psychiatric outcomes based on different groups of tobacco-product use. A 2016 study measured outcomes between four different groups based on tobacco-product use (non-use, e-cigarette use only, conventional cigarette uses only, and dual-users) [18]. The main findings included that dual-users had the most severe and pervasive comorbidity, followed by single product users and non-users respectively. A similar study found that e-cigarette users were at an intermediate risk status between non-users and dual users of tobacco products [19]. These findings depict a strong link between e-cigarette use and mental health issues. This is something that is not cautioned during advertisements nor often considered by populations when partaking in this habit.

The mechanism of causality between nicotine (contained in many e-cigarettes) and psychosis has further been studied [20]. We are aware that the mechanism of action of antipsychotics is dopamine-2 receptor antagonism and that a well-known state in psychosis is a high dopamine level within the mesolimbic dopaminergic pathways in the brain. It is also well known that GABA is an inhibitory neurotransmitter while glutamate is excitatory. Nicotine stimulates dopamine release in areas throughout the brain (such as within the nucleus accumbens, which leads to the perception of pleasure and rewards) [21]. Nicotine also increases GABA and glutamate neurotransmission; however, the overall effect is actually decreased GABA and increased glutamate neurotransmission. This leads to an overall excitatory state which further prolongs dopamine release. A similar mechanism (excess glutamate leading to activation of dopaminergic neurons) has been implicated in psychosis, which may explain the link between nicotine/e-cigarette use and psychosis.

A recently published case report described a 19-year-old Caucasian patient who suffered from acute psychosis after abrupt withdrawal from nicotine vaping [22]. The patient was using e-cigarettes, with increasing frequency, for six to nine months and had no previous psychiatric history. He then quit use abruptly and developed acute agitation and auditory hallucinations. These symptoms stopped when he received a nicotine patch. This case is somewhat similar to our patient of study who was not using e-cigarettes when she was admitted with acute psychosis. Perhaps it was due to nicotine withdrawal. Both the above case and our patient of the study were young, Caucasian, and had no prior psychiatric history. They both also had a negative urine drug screen (positive for THC only) on admission, unremarkable labs, and the development of overt hallucinations.

Our patient of the study had grandiose delusions and delusions of being sexually abused. She was also very paranoid, had auditory and visual hallucinations and erratic behavior (including symptoms of hypersexuality). However, after treatment with antipsychotics (D2-receptor antagonism), her symptoms reverted over a period of three weeks, and she went back to her pre-morbid state. It is possible that the e-cigarette our patient smoked contained nicotine which, due to the above mechanism, precipitated/caused an acute psychotic episode in combination with her other possible triggers: cannabis use, a pineal gland cyst, and the proinflammatory state from e-cigarette associated lung injury and/or COVID-19. The proinflammatory mechanism triggering psychosis is reinforced by the CDC's recently published evidence on e-cigarette associated lung injury [17].

Pineal cyst and unexplained hypertension as predisposing factors for anxiety and subsequent cannabis use and psychosis 

The pineal gland is a small endocrine gland located between the cerebral hemispheres, and its main function is to secrete the hormone melatonin. The pineal gland's functions include the production of melatonin and the modulation of sex hormones [23]. Melatonin is vital for the regulation of the circadian rhythm, and, thus, dysfunction of this gland can cause an array of clinical problems, including insomnia. Due to its location, an enlargement of this gland (due to a tumor or cyst) can also lead to compressive symptoms such as hydrocephalus, impaired upward gaze (Collier's sign), and even precocious puberty.

In recent years, there has been a rising incidence of patients with pineal gland lesions presenting with dysregulation of sex hormones and the first episode of acute psychosis. In our case, a 21-year-old Caucasian with a pineal gland cyst presented similarly. She had marked sleep disturbances and hyper-sexuality, which could be explained by the cystic malformation of this vital gland. Pineal tumors are more common in Asian countries than in Western countries [24]. This is largely due to an increased incidence of germ cell tumors in this population. In contrast, asymptomatic pineal cysts are usually an incidental neuroimaging finding (this was the case in our patient of study). Of note, in a retrospective study of 1,000 consecutive magnetic resonance images (MRIs), true pineal cysts (5 mm or larger in diameter) were found in 0, 1.8, and 2.6 percent of children ≤12 years of age, teenagers, and adults, respectively [25]. They appear hypodense with respect to cerebrospinal fluid, may or may not enhance with contrast, and calcifications are found within the wall of approximately half of all cases. From this, we note that the incidence of such cysts increases with age, and the characteristics of symptomatic cysts are different than those that are asymptomatic. The natural history of a pineal gland cyst can vary - most remain silent for years, and some may spontaneously collapse [25]. However, symptomatic pineal cysts are usually larger than those that are found incidentally, and their incidence is highest in young women between 21 to 30 years of age [26]. This was the case with our female patient, whose age fell within this expected age range. Symptoms of pineal cysts are similar to those described above and are typically caused by aqueductal compression.

There is research available that suggests that pineal gland cysts can remain asymptomatic or can lead to a variety of psychiatric symptoms such as anxiety or precipitate the first episode of psychosis. Our patient presented with her first psychiatric admission with a high functioning pre-morbid state and without any significant past medical history. Her symptoms included anxiety, difficulty sleeping, auditory and visual hallucinations, paranoid delusions regarding sexual abuse, hypersexual behavior, and suicidal ideation with dysphoric mood. The pineal gland cyst was not known; however, it is now understandable that it may have led to the patient's difficulty sleeping due to dysfunctional melatonin production.

During our patient's admission, she underwent brain imaging (MRI) which revealed the pineal gland cyst. The lesion was described as "hyperintense, measuring 1.3 cm x 1.3 cm." Our patient was also found to have an occipital venous anomaly, but the remainder of the imaging was normal. Her previous CT head depicted normal brain white matter, no mass effect, and no hemorrhage. Neurosurgery was consulted, due to her MRI findings, to assess if these rather incidental findings could explain our patient's first presentation of psychosis. Our patient of study also developed unexplained hypertension (which could indicate a form of endocrine dysfunction, which pineal gland dysfunction is known to cause). Neurosurgery's impression was that the findings were incidental but suggested that a repeat brain MRI in three-six months was required to follow up on the cyst.

The pathophysiology of psychosis in patients with pineal lesions is debated. An interesting theory is that it is due to the dysfunctional production of melatonin and the chain of events thereafter. Melatonin production in the pineal gland occurs from serotonin via an enzyme called methyl-transferase. If this enzyme is dysfunctional, melatonin is not produced, and instead, alkaloids are overproduced in the body. These alkaloids are hallucinogenic and may precipitate hallucinations in some patients. Another hypothesis is based on the finding that intravenously administered melatonin produced rapid and marked exacerbation of hallucinations in schizophrenic patients and that compounds (such as cocaine, L-dopa, and amphetamines) that produce syndromes similar to psychosis actually stimulate an increase in melatonin secretion [27].

Furthermore, existing case reports further corroborate the above link. A case from 2010 was based on an adolescent male who initially developed subthreshold symptoms of psychosis which progressed to frank psychosis [23]. He was treated with antipsychotics and psychotherapy, however, was unresponsive to both. Eventually, he underwent routine neuroimaging and was found to have a 29 mm pineal tumor (mixed germ cell histology) with hydrocephalus on an MRI scan. In contrast to our case, this male patient had a subthreshold prodrome for approximately four years which is significantly longer than our patient's psychiatric decline that reportedly occurred over the course of a few weeks. Furthermore, our patient's pineal cyst was much smaller (13 mm in maximum dimension). However, in terms of symptoms, there were similarities: the patient from the aforementioned case had unusual behaviors, symptoms of obsessive-compulsive disorder (OCD), which progressed to overt psychosis such as olfactory and auditory hallucinations, thought withdrawal, and a delusion surrounding "mind-reading". Our patient also had a prodrome, albeit shorter, where she reported increased anxiety before progressing to overt psychosis.

Perhaps the rate of pineal tumor growth or overall gland size can impact the duration of the psychotic prodrome. Of note, once the other patient's pineal lesion was found, his antipsychotics were immediately stopped, and he was commenced on a six-month high-dose chemotherapy regimen. His pineal tumor decreased from 29 mm to 7 mm at the conclusion of therapy, and his psychotic symptoms remitted to the "mild range" that consisted of some paranoia, mild unusual thought content, and perceptual abnormalities [23]. His symptoms no longer reached the psychotic threshold. He also returned to public high school to attend class (although he had motor difficulties and mild depression). His overall mental health status and social functioning improved significantly, and no recurrence of psychosis occurred since the onset of chemotherapy.

This study highlights that a large pineal tumor may be involved in the pathogenesis of psychosis and that a reduction in tumor mass can lead to the resolution of psychosis. Our patient responded significantly to antipsychotic medication, and due to the nature of her pineal lesion, no chemotherapy was indicated. Thus, one may hypothesize that patients with less aggressive and relatively benign lesions may be more responsive to psychopharmacology than those with more aggressive/malignant lesions.

From the above, it may seem reasonable to consider neuroimaging in young patients presenting with first-episode psychosis (in addition to the routine detailed psychiatric and medical history and drug screen). This may be important to rule out any organic causes of psychosis so that correct management can occur promptly to reduce morbidity. In addition to brain imaging, cerebrospinal fluid (CSF) analysis in the work-up of organic psychosis is obtained in some cases. We mentioned ruling out NMDA encephalitis as an organic condition presenting with psychotic symptoms. Due to lower clinical suspicion for anti-NMDA encephalitis, a lumbar puncture for CSF analysis was deferred. Per neurology, a lumbar puncture was not indicated. CSF testing is more sensitive than serum analysis of antibody titers, an important consideration for clinicians facing this diagnostic dilemma in first break psychosis patients. One study identified that 13.2% of cases did not have detectable serum antibodies but were always present in CSF [28]. It appears this continues to be rather controversial, with recommendations for either serum screening for antibodies only or both serum and CSF analysis [29]. 

Continuing our discussion of radiologic findings, a retrospective study on structural neuroimaging of young patients with first-episode psychosis was published in 2016 [30]. Thirty-two patients aged 18-48 years were admitted with first-episode psychosis and had either a CT or MRI scan of the brain. Thirty-seven percent of these patients had "incidental brain findings" such as arachnoid cysts, brain atrophy, lateral ventricle anomalies, which were "not thought to be causally related to psychosis". Many studies have come to the same conclusion that structural brain lesions account for <2% of psychosis, and, thus, there is minimal clinical utility in neuroimaging in young patients with first-episode psychosis. A clinical neurological examination is a more valuable starting point.

However, we note that the sample size of these studies is often small, and such conclusions are derived when pineal lesions were not found. Perhaps there should be more emphasis and widespread knowledge on the typical presenting features of pineal lesions, the typical age of presentation, and its link with possible psychosis in order to request neuroimaging when clinical suspicion arises.

The size of the normal pineal gland without any tumor or cyst can also be relevant. A study was conducted in 2015 which investigated the link between pineal gland volume in patients with schizophrenia and mood disorders [31]. The pineal gland volume of 80 patients (including 16 cases of unipolar depression, 17 cases of bipolar disorder, 17 cases of schizophrenia, and 30 controls) was measured via MRI, and the total mean pineal volume of each group was compared. The significant findings were that patients with schizophrenia had significantly decreased pineal gland volume in comparison to healthy individuals. In contrast, the pineal gland volume was not decreased in the subset of patients with mood disorders. This study reinforces the link between the pineal gland (and its deviation from normal) and psychiatric morbidity.

We learn that the pineal gland can disrupt the normal functioning of the patient. This is due to simpler mechanisms such as melatonin synthesis disruption leading to sleep disturbance but also more complex, less understood mechanisms such as increased production of vinca-alkaloids precipitating acute-onset psychosis. Future research could attempt to measure levels of these alkaloids and contrast them with the onset of psychosis. The existing research is vast but certainly strengthens the relationship between pineal gland dysfunction and neuropsychiatric symptoms. Our case contributes to this link.

Adverse reaction for the second generation of antipsychotics - hypersexuality

Sexual dysfunction in patients with schizophrenia may be related to the disease itself, psychosocial factors, somatic health, and the use of psychotropic medications [32,33]. The neurophysiological mechanisms that regulate sexual desire remain only partly understood. The interaction between brain monoaminergic (i.e., adrenergic and serotoninergic) receptors and sex hormones (i.e., testosterone) is considered pivotal for sexual responses and behaviors. Enhanced dopaminergic neurotransmission is typically associated with sexual excitation, while enhanced serotonergic neurotransmission with sexual inhibition [34-36].

Secondary drug-induced hypersexuality due to dopamine-enhancing medications like antiparkinsonian drugs is well-known and has been associated with enhanced activation of the ventral striatum, cingulate, and orbitofrontal cortices, which are similar to those areas of the brain that are related to primary hypersexual disorders [34,37].

An interesting report discusses hyper-sexual disorder where one can use the compulsivity and impulsivity model as a way to understand increased sexuality as a coping mechanism when experiencing anxiety or depression [38]. Although in our patient, this was precipitated by a suspected offending agent due to the timeline and resolution of symptoms upon cessation of the atypical antipsychotic, the case highlights that hypersexual disorder is not always associated with another primary psychiatric disorder. We suggest that is the case in our patient, who may have suffered an atypical response to medication rather than experiencing sexual preoccupations as a symptom of a primary psychiatric disorder, such as mania for which she did not meet the criteria.

As there are only a few reported clinical cases of second-generation antipsychotic (SGA)-induced hypersexuality in the medical literature, there are yet no neurobiological or imaging correlates of SGA-induced hypersexuality [34,39]. Nevertheless, an SGA-mediated increase in dopaminergic neurotransmission, a blockade of serotoninergic neurotransmission, or a combined effect, have been suggested as possible pathways for SGA-induced hypersexuality.

Several possible mechanisms have been proposed to explain the SGA-mediated hypersexuality, including an SGA-induced increase in dopaminergic neurotransmission, a blockade of serotonergic neurotransmission, or a combination of both. Thus, several cases of hypersexual disorders caused by SGA like aripiprazole, with partial agonistic effects at dopamine D2 receptors, have been reported [40,41]. Partial D2 agonists increase dopaminergic activity in the mesolimbic system, causing irregular sexual excitation. Serotonin blockade may similarly increase dopamine activity in the mesocortical-dopamine pathways [34]. However, olanzapine mediates its effect by anti-dopaminergic, anti-serotonergic, and anti-α1-adrenergic activity. The serotonergic blockade results in an increase in dopamine activity in the mesocortical dopamine pathways and is considered a possible explanation for hypersexuality in patients taking olanzapine [34]. Another postulated mechanism is that olanzapine-mediated α1-adrenergic and anticholinergic antagonism in the peripheral nervous system may also affect erectile, orgasmic, and ejaculatory function, with several cases of olanzapine-induced priapism and spontaneous ejaculations reported [34,42,43]. Interestingly, a randomized control trial (RCT) of 42 schizophrenic patients assessing for impairment in sexual function concluded that switching from first to second-generation antipsychotics improved sexual dysfunction [44].

Successful recovery on first-generation antipsychotic and mood stabilizer

As of 2012, there were eighteen antipsychotic medications approved for schizophrenia and/or psychosis, with at least ten atypical agents available in 2016 and a new FDA approval in 2019 totaling eleven atypical antipsychotics [45,46]. There are about 20 antipsychotics in addition to long-acting injectables available for use in the United States. Clinical practice guidelines aid clinicians in determining the antipsychotic of choice by careful consideration of anti-dopaminergic potency, actions on additional receptors, and resulting side effect profiles used in the analysis of risks vs. benefits. There is varying literature on the use of one generation over the other or even efficacy on agents in the same class. The psychopharmacologic treatment in our case fluctuated, as did the patient's course.

Haloperidol, a typical high-potency agent, was also utilized for as-needed doses for agitation. The challenge our team faced was the question of her worsening psychosis as evidenced by bizarre behavior, or was this also a case of neuroleptic-induced slowing and a negative effect of pharmacological intervention? When our patient was then cross-tapered to olanzapine, a second-generation agent used to stabilize acute presentations on inpatient units, and failed to show any signs of stabilization of severe symptoms and developed side effects, we reverted back to haloperidol.

Haloperidol's benefits on positive symptoms of schizophrenia are clearly due to higher potency D2 blockade. There is evidence of even ultra-low doses (1 mg) being beneficial in first break psychosis [47]. Our patient did not require higher than 20 mg to improve symptoms. However, when reviewing the literature, it is clear there is little evidence to support the first-line use of this agent. An RCT revealed that the atypical antipsychotics such as aripiprazole, risperidone, and olanzapine were more effective in first break psychosis compared to haloperidol [48]. Haloperidol also had a high dropout rate of 89.28%. A 2014 review mentioned haloperidol was not superior to lower potency agents in terms of response [49]. Another review showed limited evidence that haloperidol is alleviating positive symptoms of schizophrenia more compared to other antipsychotics and concluded there is no evidence to suggest one typical agent is superior to the other [50]. An older clinical trial suggested considering olanzapine to haloperidol as a first-line agent in first break psychosis [51], an agent that ultimately failed in our case.

Our patient recovered on a typical high-potency antipsychotic in combination with a mood stabilizer, valproic acid. Valproic acid is an antiepileptic agent often utilized by psychiatrists in the treatment of mental disorders, most notably bipolar spectrum disorders. There is evidence of its complex neuroprotective properties [52]. There is limited evidence in the literature to support valproate use in psychosis. Treatment is usually reserved for severe cases marked by significant agitation/aggression. A 2016 meta-analysis revealed the benefits of valproate augmentation in combination with antipsychotic medication [53]. Similarly, a 2016 review from the Cochrane database reported a positive treatment response when using valproate with an antipsychotic compared to placebo, in addition to noting the effect on excitement and aggression [54]. It is, therefore, reasonable to consider this mood stabilizer in both affective and non-affective psychosis, as there was some benefit in our patient.

A bipolar spectrum disorder was unlikely in the absence of major criteria for a manic or hypomanic episode. The patient's behavior was disorganized, consistent with psychosis, and not better explained by behavioral indiscretion in mania. Throughout admission, she did not exhibit any flight of ideas, talkativeness, or pressured speech and instead had severe thought blocking. There was the absence of increased goal-directed activity or decreased need for sleep. The patient was severely psychotic and internally preoccupied, without obvious distractibility seen in mania. Few grandiose comments delusional in nature, often seen in bipolar mania with psychotic features, were present. However, the patient's affect was incongruent and neither elated nor expansive. She experienced significant lability early in admission. Affective lability is also seen in psychotic spectrum disorders, though more familiar to clinicians in the setting of bipolar spectrum disorders. A recent study identified no significant differences in affective lability between bipolar I disorder vs. schizophrenia patients [55].

COVID-19 illness - new disease, can it cause psychosis?

Psychosis caused by viral infection has been associated with other novel infectious disease outbreaks. Respiratory viruses have been associated with psychosis since the 1918 influenza pandemic when Menninger published a report of 100 patients with neuropsychiatric sequelae associated with influenza infection [56,57]. A link between psychosis and other strains of coronavirus has been known for a long time, including the strain that causes severe acute respiratory syndrome (SARS-CoV) [58-60]. Some of the frequent symptoms observed in SARS-related psychosis include persecutory delusions, auditory and visual hallucinations, delusions of grandeur, elated mood, suicidal ideations, disorientation, and sleep disturbances [58,61]. There is even mania associated with COVID-19 infection published in the literature [60].

Neurological and psychiatric sequelae of COVID-19 are actively being studied. A recent review summarized COVID-19 neurological sequela, ranging from olfactory and gustatory dysfunction and other cranial nerve impairments, headaches, cognitive, memory, and sleep changes, fatigue, akinetic mutism, stroke, worsening of dementia, and Gillian-Barre syndrome [62]. Anxiety, depression, posttraumatic stress disorder (PTSD), somatization, OCD, and suicide were associated with psychiatric effects during the pandemic [63].

The theory that viral agents are etiologically linked to psychosis has gained support in recent years. Studies have found that patients with new-onset psychosis have significantly higher serum antibody titers for viruses like Epstein-Barr virus (EBV), herpes simplex virus type 1 (HSV1), cytomegalovirus (CMV), mumps, measles, varicella-zoster, and Japanese encephalitis virus [64-66]. Several possible pathological mechanisms have been postulated to explain the neurocognitive and psychotic sequelae of COVID-19. One of the widely popular mechanisms is the direct viral infiltration of the central nervous system (CNS), as has been demonstrated by the neuroinvasive potential of many strains of the coronavirus. It is believed that coronaviruses can migrate from the respiratory tract to the brain via retrograde axonal transport from the olfactory bulb or by hematogenous dissemination into the CNS [56,65]. In studies on mice, the human respiratory coronavirus strain OC43 (HCoV-OC43) is shown to have a preferential tropism for infecting neurons versus other neural cells (e.g., oligodendrocytes, astrocytes, microglia) [58,67]. After entering the CNS, CoV has been shown to induce neuronal cell death in mice. Further, mice infected with HCoV-OC43 develop chronic encephalitis, marked by viral persistence in neurons and behavioral abnormalities [68]. These findings demonstrate that respiratory viruses like coronavirus can be potentially neuroinvasive and cause neurocognitive problems and behavioral problems, including psychosis.

The second widely suggested mechanism for COVID-related psychosis involves cytokine network dysregulation. The main receptor that SARS-CoV attaches to for intracellular invasion is angiotensin-converting enzyme 2 (ACE2), which is expressed in both neurons and glia. In experimental studies of intranasally-inoculated SARS-CoV-1 infection in ACE2 transgenic mice, upregulation of proinflammatory cytokine secretion (e.g., tumor necrosis factor-alpha [TNF-alpha], interleukin-1 beta [IL-1-beta], Interleukin 6 [IL-6]) by neurons and astrocytes was observed [63]. In addition to this, peripheral increase in proinflammatory cytokines production in response to COVID-19 infection can lead to increased permeability and compromised integrity of the blood-brain barrier [67,69].

Some other possible mechanisms of psychosis in COVID-19 patients have also been proposed, like post-infection autoimmunity, gut microbial alterations, treatment with immunomodulatory drugs, and social isolation due to strict quarantine and social distancing measures in the wake of the COVID-19 pandemic [59,67].

Another highlight of this patient's case related to prior COVID-19 infection is the discussion of hypertension. It is unclear if the patient's hypertension was solely due to psychomotor agitation. We ruled out common secondary causes such as renal structural abnormalities. There is evidence of enhanced angiotensin II signaling driven by SARS-CoV-2 infection, which affects the renin-angiotensin system and may lead to hypertension [70].

Our patient likely had COVID-19 in March 2020 as she had an illness back then. Later during her hospitalization, one year later, her antibodies were positive for COVID-19, as she got tested for them before getting a vaccine. She received the first vaccine in early April 2021. It could be possible that in combination with her other triggers, such as cannabis use and a pineal gland cyst, a reactivation of the viral lesion in the neural cells occurred.

## Conclusions

Clinicians continue to face challenges in first break psychosis cases regarding diagnosis and treatment. Medical conditions such as infections with novel viruses and increasing potency of highly psychoactive substances require careful investigation and consideration as the potential cause for these episodes. Psychopharmacological treatment of psychosis varies from individual to individual, each with its own risk of side effects that can extend hospitalization if serious complications occur. It may be difficult to differentiate such side effects from a symptom of schizophrenia spectrum disorders. Haloperidol use is warranted for severe psychosis with careful consideration of a mood-stabilizing agent such as valproic acid. Continued research on the predictability of schizophrenia spectrum disorders after an episode of acute psychosis is warranted to aid diagnostic clarity and guide future treatment decisions.
